# Engineered Electrospun Polyurethane Composite Patch Combined with Bi-functional Components Rendering High Strength for Cardiac Tissue Engineering

**DOI:** 10.3390/polym11040705

**Published:** 2019-04-17

**Authors:** Mohan Prasath Mani, Saravana Kumar Jaganathan, Ahmad Athif Faudzi, Mohd Shahrizal Sunar

**Affiliations:** 1 School of Biomedical Engineering and Health Sciences, Faculty of Engineering, Universiti Teknologi Malaysia, Skudai 81310, Malaysia; mohanprasathutm@gmail.com; 2 Department for Management of Science and Technology Development, Ton Duc Thang University, Ho Chi Minh City, Vietnam; 3 Faculty of Applied Sciences, Ton Duc Thang University, Ho Chi Minh City, Vietnam; 4 IJNUTM Cardiovascular Engineering center, School of Biomedical Engineering and Health Sciences, Faculty of Engineering, Universiti Teknologi Malaysia, Skudai 81310, Malaysia; 5 Centre for Artificial Intelligence and Robotics, Universiti Teknologi Malaysia, Kuala Lumpur 54100, Malaysia; athif@utm.my; 6 School of Electrical Engineering, Faculty of Engineering, Universiti Teknologi Malaysia, Johor Bahru 81310, Malaysia; 7 Media and Game Innovation Centre of Excellence (MaGICX), Institute of Human Centered Engineering (iHumEn), Universiti Teknologi Malaysia, Skudai 81310, Malaysia; shahrizal@utm.my; 8 School of Computing, Faculty of Engineering, Universiti Teknologi Malaysia, Skudai 81310, Malaysia

**Keywords:** polyurethane, basil/titanium dioxide, physico-chemical properties, biocompatibility, cardiac tissue applications

## Abstract

Cardiovascular application of nanomaterial’s is of increasing demand and its usage is limited by its mechanical and blood compatible properties. In this work, an attempt is made to develop an electrospun novel nanocomposite loaded with basil oil and titanium dioxide (TiO_2_) particles. The composite material displayed increase in hydrophobic and reduced fiber diameter compared to the pristine polymer. Fourier transform infrared spectroscopy results showed the interaction of the pristine polymer with the added substances. Thermal analysis showed the increased onset degradation, whereas the mechanical testing portrayed the increased tensile strength of the composites. Finally, the composite delayed the coagulation times and also rendered safe environment for red blood cells signifying its suitability to be used in contact with blood. Strikingly, the cellular toxicity of the developed composite was lower than the pristine polymer suggesting its compatible nature with the surrounding tissues. With these promising characteristics, developed material with enhanced physicochemical properties and blood compatibility can be successfully utilized for cardiac tissue applications.

## 1. Introduction

Cardiovascular diseases (CVDs) are estimated to claim 17.9 million deaths every year which is 31% of all global deaths [1]. The market for cardiovascular repair devices is increasing day by day and is expected to reach USD 4.5 billion by 2025 [2]. Myocardial infarction is a cardiac disease which leads to heart failure and has been the major cause of death worldwide for the past two decades [3,4,5]. Owing to poor regenerative capability of myocardium and the formation of scar around the infected site, the regeneration of damaged heart tissue is still challenging [3,6,7]. The commercially available products with cardiovascular applications are autografts, allografts, and xenografts. They were limited in the treatment of CVD owing to scarcity of donor site and high immunogenic response [8]. In replacement of large-diameter blood vessels (>6 mm), expanded polytetrafluoroethylene (ePTFE) or Dacron (polyethylene terephthalate fiber), have been used. However, the small-diameter synthetic grafts showed a high rate of failure due to thrombus formation [8,9]. Hence, there is a continuous search for new materials with advanced properties. Recently, tissue engineering has become a promising approach for cardiac regrowth through fabricating scaffolds based on biomaterials to support the cellular in-growth [3]. Further, the scaffolds used for the cardiac tissue engineering must resemble the native cardiac extra cellular matrix (ECM) structure. Myocardium ECM provides a nanofibrous microenvironment which aids the cardiac cell adhesion and ingrowth. It was reported that the scaffold developed using polymeric materials can mimic the native ECM of myocardium [10,11,12]. Many techniques have been emerged to convert the polymer structures into nanofibrous to mimic the myocardium ECM structure. The widely used techniques for fabricating the fibrous scaffold are self-assembly, drawing, phase separation, template synthesis, and electrospinning [13]. 

**Figure 1:**
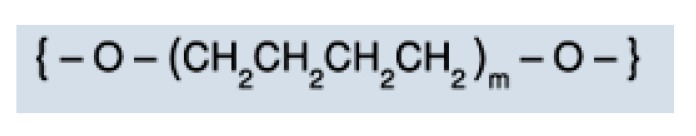
Chemical structure of polyether urethane.

In this research, electrospinning is used to fabricate the nanofibrous scaffold. Electrospinning has been regarded as a versatile and well-established method to fabricate the fibrous scaffolds. It could be able to produce fibers with diameter ranging from micrometers to nanometers [14]. It has three operational parameters (voltage, flow rate, and distance) and it could be manipulated to control the fiber diameter, their orientation, and density [3,15]. The nanofibers obtained through electrospinning technique possess promising characteristics like high surface area with an interconnected porous structure [13,16]. Further, the electrospun nanofibers have similarity with the native ECM structure which can support the cardiac cells growth [17,18,19]. The biomimetic scaffolds can be developed from natural materials or synthetic polymers. The various natural and synthetic polymers used in the tissue engineering applications were collagen, alginate, chitosan, poly(glycolic acid), poly(lactic acid), poly(p-dioxanone), and poly-co-glycolide [20]. Polyurethane (PU, medical grade polyether polyurethane) was used to fabricate the cardiac patch and their chemical structure was given in Figure 1. PU was selected for this study because of its biodegradability and good biocompatibility behavior [21]. Further, it possesses good oxidation and temperature stability properties [22]. Owing to these desirable properties, polyurethane used in widespread applications in tissue engineering.

The scaffolds used in the cardiac tissue engineering must reduce the thrombus formation and also possess good mechanical strength to support the cardiac tissue growth [23]. Recently, it was reported that the addition of essential oils in the electrospun scaffold reduced the thrombus formation suggesting the improved blood compatibility [8,24]. Further, some literatures have reported that the presence of metallic particles in the electrospun scaffold exhibited improvement in the tensile strength [25,26]. Hence, this research aims to use basil oil and titanium dioxide (TiO_2_) and analyze their combination effect for cardiac tissue engineering. Basil is widely found in tropical area of the world from central Africa to southeast Asia. The oil form the basil contains various chemical components in which high concentrations of linalool and methyl chavicol at a ratio of 3:1 are reported. Other bioactive constituents present were 1,8-cineole, eugenol, and myrcene. The aroma components present in the basil includes 1,8-cineole and methyl eugenol [27]. Further, the chemical constituents present in the basil oil are reported to have antimicrobial and antioxidant effects [28]. Metallic biomaterials are found to be widely used in medical applications because of their high strength and resistance to corrosion. TiO_2_ and other titanium-based alloys possess better biocompatibility, osseointegration, high corrosion and wear resistance, and high strength. More, Ti has a high melting point, boiling point and elastic modulus. For scaffold to be used in the cardiac patch application, it must possess sufficient strength. Hence, a Ti with high strength and elastic modulus might provide good strength to the developed patch [29]. The aim of the study was to fabricate and characterize an electrospun cardiac patch based on polyurethane added with basil and TiO_2_. 

## 2. Experimental

### 2.1. Materials

PU (medical grade polyether polyurethane) was supplied from Lubrizol, Wickliffe, United States. Basil oil was obtained from AEON, Johor, Malaysia. Titanium dioxide was supplied from Sigma Aldrich, Gillingham, UK. Dimethylformamide (DMF) was purchased from Merck, Burlington, New Jersey, US. All reagents used in the blood compatibility studies were obtained from Diagnostic Enterprise, Thiruvananthapuram, India.

### 2.2. Preparation of PU and Composite Solutions

To make 9 wt% of PU homogenous solution, 0.270 g of PU was dissolved 3 mL of DMF and stirred overnight. Similarly, 4 *v*/*v* % of homogenous basil solution was done by adding 80 µL of basil oil to 2 mL of DMF and stirred for 1 h maximum. Further, 4 wt % of TiO_2_ homogeneous solution was prepared by adding 0.080 g of TiO_2_ in 2 mL of DMF and stirred for 2 h. To obtain PU/basil solution at a ratio of 8:1 *v*/*v*%, the prepared basil solution (4 *v*/*v*%) was added to the PU homogenous solution (9 wt %) and stirred for a maximum of 2 h. Similarly, the solution of PU/basil/ TiO_2_ with a ratio of 8:0.5:0.5 *v*/*v*% was attained by adding the prepared basil solution (4 *v*/*v*%) and TiO_2_ solution (4 wt %) to the PU homogenous solution (9 wt %) and stirred for a maximum of 2 h.

### 2.3. Electrospinning of PU and Composite Solutions

In this research, electrospinning apparatus (Progene Link Sdn Bhd, Selangor, Malaysia) was utilized to fabricate the fibrous scaffold. The collector drum used was static and grounded. All scaffolds were electrospun at constant optimized parameters. The distance between needle tip to collector distance and flow rate were fixed at 20 cm and 0.3 mL/h, respectively. The applied voltage was 11 kV and was performed at room temperature. After fibers collected on the aluminum oil, it was dried under vacuum to remove any residual DMF.

### 2.4. Physico-chemical Properties

#### 2.4.1. Field Emission Scanning Electron Microscopy (FESEM)

Morphology of the electrospun nanofibers were examined using FESEM unit (Hitachi SU8020, Tokyo, Japan). Prior to scanning, a small piece of electrospun nanofibers were gold coated and captured at different magnifications. Image J software was used to measure the fiber size of the electrospun membranes by measuring 30 locations randomly.

#### 2.4.2. Fourier Transform Infrared (FTIR) Spectroscopy Analysis

FTIR spectroscopy was conducted using a Nicolet is 5 spectrophotometer (Thermo Fischer Scientific, Waltham, MA, USA) to determine the chemical changes of the electrospun membranes. The Zinc Selenium (ZnSe) is used as an ATR crystal. The spectra were inspected at wavenumber range of 600–4000 cm^−1^ in room temperature at a resolution of 4 cm^−1^.

#### 2.4.3. Contact Angle Measurements

To monitor the wettability of the scaffolds, static contact angle was measured by video contact angle equipment (AST Products, Inc., Billerica, MA, USA). The medium used was distilled water. A droplet (0.5 μL) of water was placed on the nanofiber surface and their static image was snapshotted using video camera. The manual contact was measured from the snapshotted image using computer integrated software and it was repeated for three trials.

#### 2.4.4. X-ray Diffraction Analysis (XRD) Analysis

To know the structural changes of the developed membranes, X-ray diffraction study was performed. The XRD pattern was obtained using XRD analyzer (Rigaku, Tokyo, Japan) for the range of 10–90° using CuK_α_ as radiation.

#### 2.4.5. Thermal Characterization

Thermal analysis was conducted in thermogravimetric analysis (TGA) unit (PerkinElmer, Waltham, MA, USA) to study the degradation behavior of the electrospun membranes. The data were obtained by applying heat to the samples (3 mg) in the temperature range between 30 °C to 1000 °C with rate of 10 °C min^−1^ and performed under nitrogen atmosphere.

#### 2.4.6. Surface Roughness Measurements

The surface roughness of the electrospun membranes were determined using a atomic force microscopy (AFM) unit (NanoWizard®, JPK Instruments, Berlin, Germany). The scanning of electrospun membranes was done under normal atmospheric conditions in size of 20 µm by 20 µm area and images were obtained in 256 resolution. From the obtained images, Ra was calculated at three different locations randomly using JPKSPM software.

#### 2.4.7. Mechanical Characterizations

The mechanical performance of the fabricated nanofibers was evaluated using uniaxial testing machine (Gotech Testing Machines, AI-3000, Taichung, Taiwan) according to ASTM D882-10. To start, the nanofibers of 40 mm by 15 mm were cut and clamped on the grip ends of the uniaxial tensile machine. Then, the clamped nanofibers were stretched at a cross head speed of 10 mm/min with a load cell of 500 N until failure. The average tensile strength was determined from the machine generated stress strain curve. 

### 2.5. Coagulation Assays

#### 2.5.1. Activated Partial Thromboplastin Time (APTT) and Prothrombin Time (PT) Assay

All prepared scaffolds were cut into a size of 1 by 1 cm^2^ and incubated with 50 µL of platelet poor plasma (PPP) for 1 min at 37 °C. For APTT assay, PPP was added with 50 µL of rabbit brain cephaloplastin reagent and incubated for 2 min at 37 °C. Then, the initiation of the blood clot was done by adding 50 µL of CaCl_2_ and the clot formation was examined with a sterile steel needle. The time taken for the blood clot was measured using a chronometer. Similarly, for PT assay, the samples incubated with PPP was added with 50 µL of NaCl–thromboplastin reagent (Factor III) which initiate the blood clot. PT is the time taken for the clot formation which was determined using chronometer [8].

#### 2.5.2. Hemolysis Assay

The toxicity of electrospun membranes with red blood cells were investigated using hemolysis assay. To begin this assay, all prepared samples with a size of 1 by 1 cm^2^ was soaked in 0.9% *w*/*v* of physiological saline at 37 °C for 30 min. Next, they were added with a combination of aliquots of citrated blood and diluted saline prepared at a ratio of 4:5 for 1 h at 37 °C. Then, the samples were removed and centrifuged at 3000 rpm for 15 min. Finally, the optical density (OD) was recorded for the aspirated supernatant at 542 nm which represents the release of hemoglobin. The percentage of hemolysis or hemolytic index was calculated using the formula [8]:

Hemolysis ratio (HR) = (TS − NC)/(PC − NC) × 100 (1)

where TS, NC, and PC are measured absorbance values of the test sample, negative control, and positive control at 542 nm, respectively.

### 2.6. Characterization of in Vitro Biocompatibility

3-(4,5-dimethylthiazol-2-yl)-5-(3-carboxymethoxyphenyl)-2-(4-sulfophenyl)-2Htetrazolium, inner salt (MTS) assay was done using human dermal fibroblast (HDF) cells to evaluate the cytotoxicity of the electrospun membranes. The fibroblast cells were grown in DMEM medium at standard conditions (37°C, 5% CO_2_). The medium was supplied with 10% FBS and was cleaned every 3 days. To start the assay, the developed membranes cut into small size and were placed in 96-well plates. The scaffold was sterilized with alcohol solution (75%) in order to support the cell adhesion. Next, the scaffold placed in each wells were seeded with confluent cells with density of 10 by 10^3^ cells/cm^2^ and cultured for 5 days. After 5 days culture, the medium was added with 20% MTS reagent and further incubated for 4 h. After 4 h, the OD was measured in spectrophotometric plate reader at 490 nm to determine the cell viability of the fabricated membranes.

### 2.7. Statistical Analysis

One-way ANOVA was performed to calculate the statistical significance (p <  0.05) for experiments with three trials and are expressed as mean ± SD. In case for qualitative experiments, an illustrative of three images is indicated.

## 3. Result and Discussion

### 3.1. SEM Investigation

**Figure 2:**
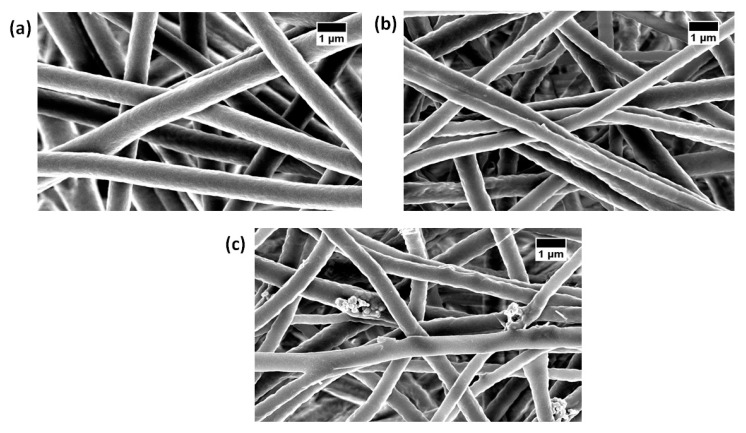
Field emission SEM (FESEM) images of (**a**) polyurethane (PU), (**b**) PU/BASIL and (**c**) PU/BASIL/TiO_2_.

**Figure 3:**
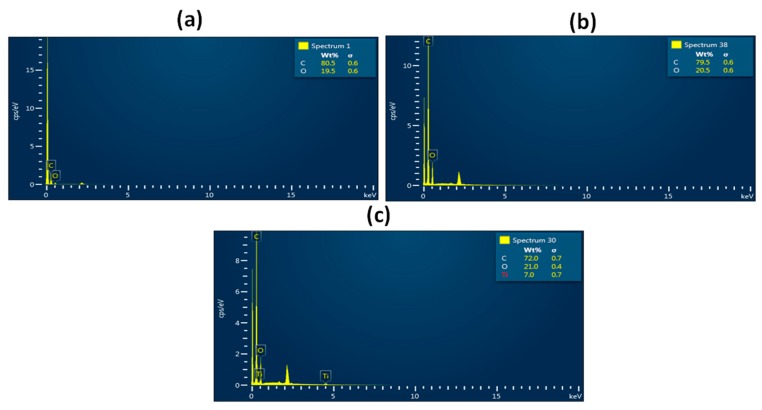
EDX of (**a**) PU, (**b**) PU/BASIL and (**c**) PU/BASIL/TiO_2_.

Morphological images of the electrospun PU, PU/basil, and PU/basil/TiO_2_ examined by field emission scanning electron microscopy are presented in Figure 2. The SEM images of electrospun membranes showed heterogeneous structure with bead-free nanofibers with random orientation. The Pristine PU showed an average fiber diameter of 870 ± 149 nm, while the PU/basil and PU/basil/TiO_2_ exhibited fiber diameters of 568 ± 153 nm and 571 ± 134 nm, respectively. It was observed that the fabricated nanocomposites showed smaller fiber diameter than the Pristine PU. PU showing a decrease in fiber diameter from adding basil which might be due to the interaction of bioactive constituents of basil oil with the PU molecules. Further, the reduction of fiber diameter was continued from adding titanium to the PU/basil, which may be due to the interaction of PU/basil oil with the titanium dioxide. Similar findings have been reported in a recent work done by Jaganathan et al. [25]. They electrospun a scaffold based on polyurethane added copper particles and the fabricated composites that showed a reduction of the fiber diameter which resembles our observation. Fu et al. fabricated a scaffold based on gelatin/polycaprolactone (PCL) and collagen/PCL for vascular tissue engineering. It was observed that the collagen/PCL showed smaller fiber diameter than the gelatin/PCL which showed enhanced cellular response [30]. Hence, our reduced fiber diameter of the fabricated composites might be suitable for the regeneration of cardiovascular tissue. Further, to confirm the presence of titanium in the polyurethane matrix, Energy Dispersive X-Ray (EDS) study was performed. The spectrum of PU and PU/basil displayed only carbon and oxygen content, while the electrospun PU/basil/TiO_2_ presented titanium content (7%) in addition to the carbon and oxygen as shown in Figure 3. Hence, EDS study confirms the presence of titanium in the polyurethane matrix.

### 3.2. Contact Angle Measurements

**Figure 4:**
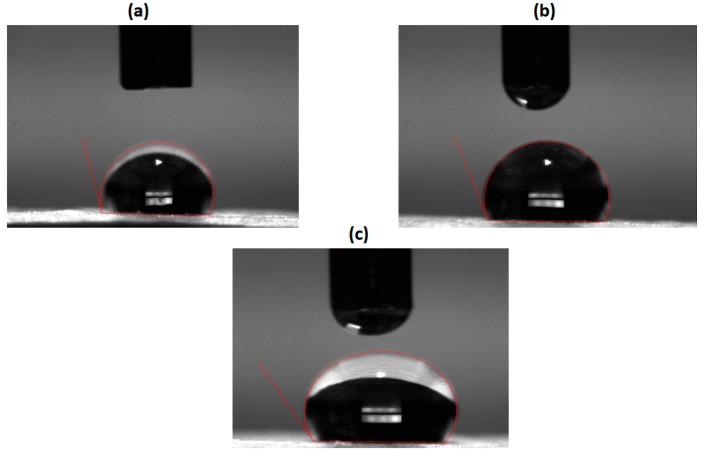
Contact angle images of (**a**) PU, (**b**) PU/BASIL and (**c**) PU/BASIL/TiO_2_.

Contact angle measurement was used to evaluate the wettability and it is a one of important property which can influence the attachment and proliferation. The contact angle images of electrospun membranes were indicated in Figure 4. The reported contact angle measurements showed a contact angle of 106 ± 3 for pure PU membrane and for PU/basil and PU/basil/TiO_2_, it was observed to be 113 ± 1 and 120 ± 2, respectively. Hence, the fabricated nanocomposites showed highly hydrophobic than the pristine PU. The influence of surface wettability (hydrophobic or hydrophilic) on the cell adhesion and proliferation was equivocal [31,32,33]. It was reported that the surfaces with moderate hydrophilicity would exhibit better cell adherence and proliferation [31,32]. On the contrary, Jaganathan et al. [33] fabricated polyurethane scaffold added with nickel oxide for the cardiac tissue engineering. It was observed that the addition of nickel oxide increased the contact angle of the Pristine PU which resembles our findings. Further, the fabricated composites showed good biocompatibility with the fibroblast cells and suggested potential candidates for the cardiac tissue engineering. Our developed composites with hydrophobic behavior might be conducive for the cardiac tissue engineering.

### 3.3. IR Analysis

**Figure 5:**
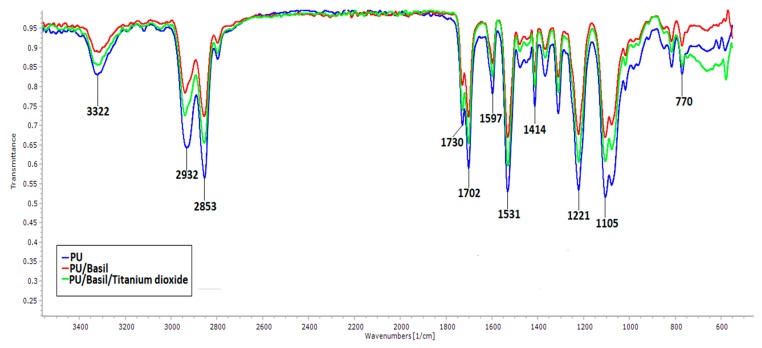
Fourier transform infrared (FTIR) spectroscopy analysis of PU, PU/BASIL and PU/BASIL/TiO_2_.

The infrared spectra for the developed electrospun scaffolds are shown in Figure 5. It was carried to examine characteristic chemical group present. Pure PU showed a broad band at 3322 cm^−1^ corresponds to NH group while the band at 1531 cm^−1^ and 1597 cm^−1^ indicates their vibration. The CH group was observed at 2932 cm^−1^ and 2853 cm^−1^ and its vibrations were located at 1414 cm^−1,^ respectively. The bands observed at 1702 cm^−1^ and 1730 cm^−1^ attributed to the CO group while the other sharp peaks at 1221 cm^−1^, 1105 cm^−1,^ and 770 cm^−1^ denoted the CO stretch respect to alcohol group [25,26]. In the both PU/basil and PU/basil/TiO_2_ scaffolds, no new peaks were observed and the observed bond groups were similar to the peaks of PU. Further, the addition of basil and TiO_2_ resulted in the decrease in peak intensity of the Pristine PU. The decrease in peak intensity was due to formation of strong hydrogen bond [34]. This was due to linking of CH and NH molecules present in the basil with the molecules of the pristine PU.

### 3.4. XRD Analysis

**Figure 6:**
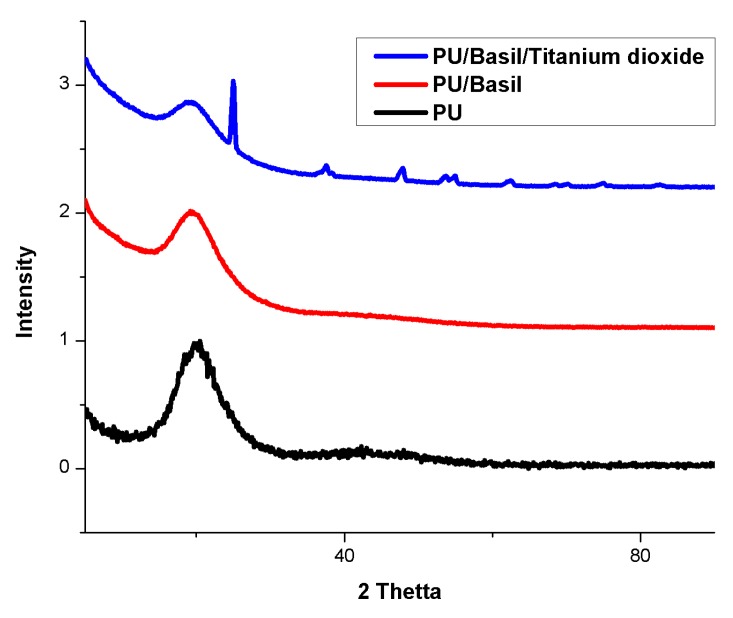
XRD images of PU, PU/BASIL and PU/BASIL/TiO_2_.

XRD studies were performed on the as-spun membranes to examine their change in crystalline behavior and obtained pattern were indicated in Figure 6. PU has 2θ at 20° which is a broad peak corresponds to its amorphous structure. In case of PU/basil, no other peaks were found and only had broad peak as that of pure PU. In the case of PU/basil/TiO_2_, some new peak was seen at 25.29°, 37.80°, 47.99°, 53.90°, 55.09°, and 62.70° along with broad spectrum at 20°. The new peak correlates with the crystal planes of TiO_2_ [35] and hence the XRD analysis confirmed the presence of metallic particles in the polyurethane matrix.

### 3.5. Tensile Properties

**Figure 7:**
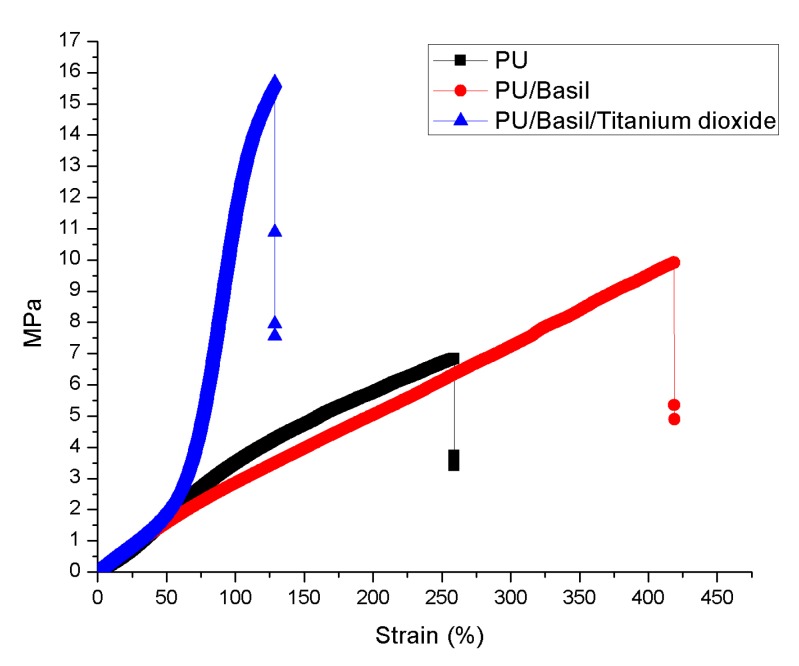
Tensile strength of PU, PU/BASIL and PU/BASIL/TiO_2_.

The tensile properties of electrospun nanofibers were characterized and the stress strain plot was shown in Figure 7. The pure PU fiber sheet showed less strength compared to the fabricated composite membranes. The Pristine PU showed a mechanical strength of 6.83 MPa, while for PU loaded with basil and basil/TiO_2_, it was increased to 9.91 MPa and 15.68 MPa respectively. The mechanical results depicted that the incorporation of basil and TiO_2_ into the polyurethane matrix resulted in the enhancement of the mechanical strength. The stated mechanical strength for native coronary arteries, human saphenous vein, and aortic values were 1.80 MPa, 3–4 MPa, and 2.6 MPa respectively [36,37,38]. The addition of basil and titanium into the polyurethane displayed better tensile values which can be exploited for the vascular applications. Jeon et al. developed polyurethane/polycaprolactone (PU/PCL) membrane incorporated with silver nitrate. It was found that the silver nitrate addition into the PU/PCL membrane resulted in the improvement of the mechanical strength which resembles with our findings [39].

### 3.6. TGA Analysis 

**Figure 8:**
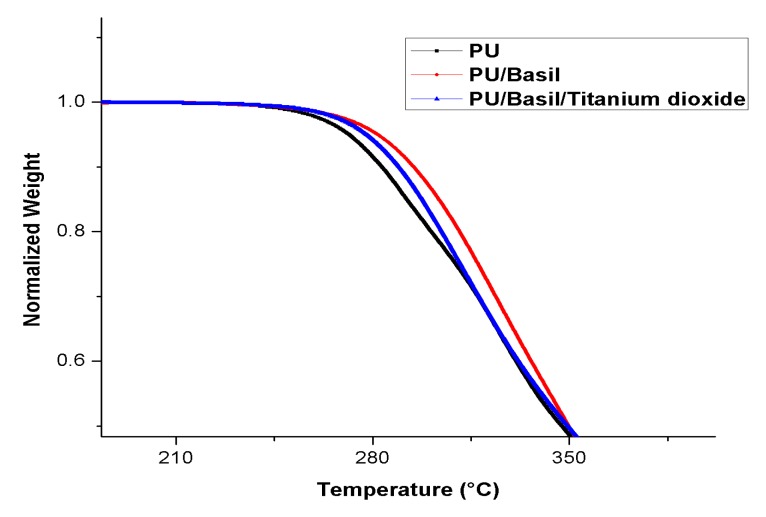
TGA of PU, PU/BASIL and PU/BASIL/TiO_2_.

**Figure 9:**
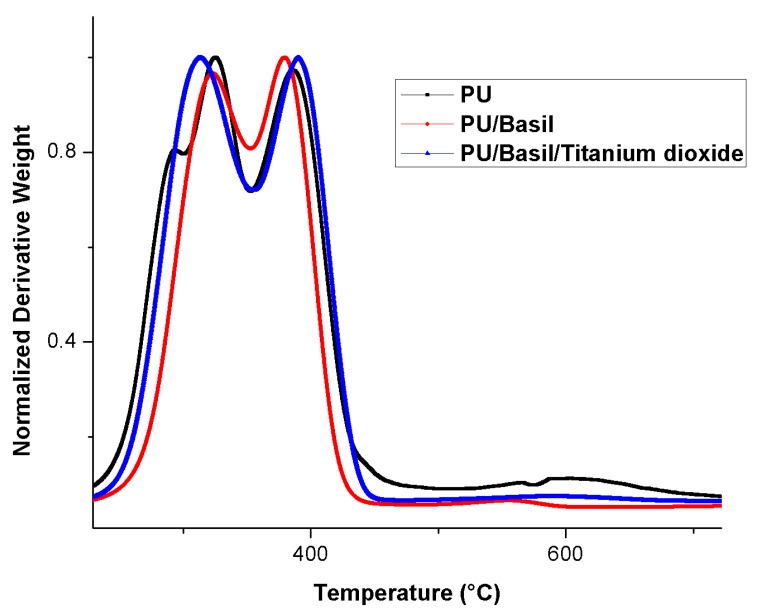
Weight residue of PU, PU/BASIL and PU/BASIL/TiO_2_.

The TGA results of the electrospun samples are displayed in Figure 8. As shown in this figure, it was observed that the initial degradation temperature of the nanocomposites was increased than the Pristine PU. The Pristine PU showed initial degradation temperature of 266 °C and for PU/basil and PU/basil/TiO_2_, it was found to 275 °C and 276 °C respectively. Hence, the thermal degradation of the pristine PU was increased with the incorporation of basil and TiO_2_. Further, the DTG curves were indicated in Figure 9 which depict the weight loss of the fabricated electrospun membranes. From the figure, it was showed that the Pristine PU showed four weight loss peaks while in electrospun PU/basil and PU/basil/TiO_2_, it had two weight loss peaks only. It was observed that the number of weight loss peaks for the pristine PU was decreased with the incorporation of basil and TiO_2_ suggesting their reduced weight loss.

### 3.7. AFM Analysis

**Figure 10:**
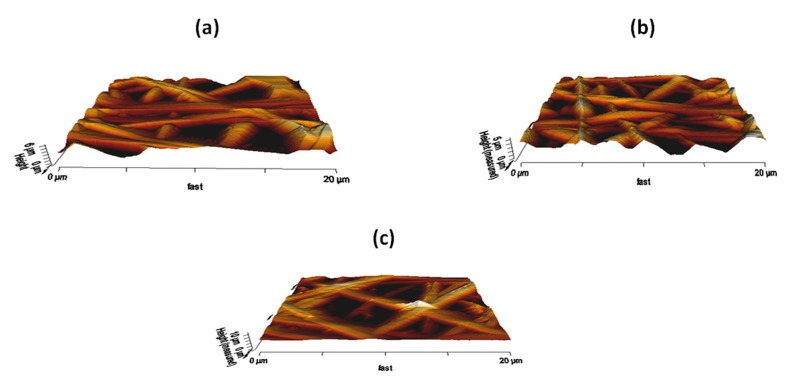
AFM images of (**a**) PU, (**b**) PU/BASIL and (**c**) PU/BASIL/TiO_2_.

AFM analysis was carried out to evaluate the surface properties of the electrospun membranes and their representative AFM images before and after basil and TiO_2_ incorporation are presented in Figure 10. The PU surface showed average roughness of 776 nm, while the PU added with basil and basil/TiO_2_ exhibited roughness of 458 nm and 928 nm respectively. The incorporation of basil resulted in the decrease in the surface roughness while adding TiO_2_ the surface roughness was enhanced compared to the pristine PU. The decrease in the surface roughness for the PU/basil composite was might be due the presence of basil oil bioactive components. However, the while incorporating TiO_2_ to the PU/basil it displayed significant improvement in the surface roughens. The presence of TiO_2_ particles in the composites may overcome the effect of bioactive constituents from basil oil resulting in the improved surface surfaces. Jaganathan et al. electrospun polyurethane scaffold added with nickel oxide. The addition of nickel oxide resulted in the enhancement of the surface roughness which correlates with our findings [33]. Parizek et al. studied the proliferation of vascular smooth muscle cells in the surface modified polyethylene. It was found that the surface modified polyethylene exhibited improvement in the surface roughness and also exhibited enhanced proliferation of vascular smooth muscle cells [40]. Our developed composites showed improvement in the surface roughness which might be suitable for the proliferation of vascular smooth muscle cells.

### 3.8. Blood Compatibility Assessments

**Figure 11:**
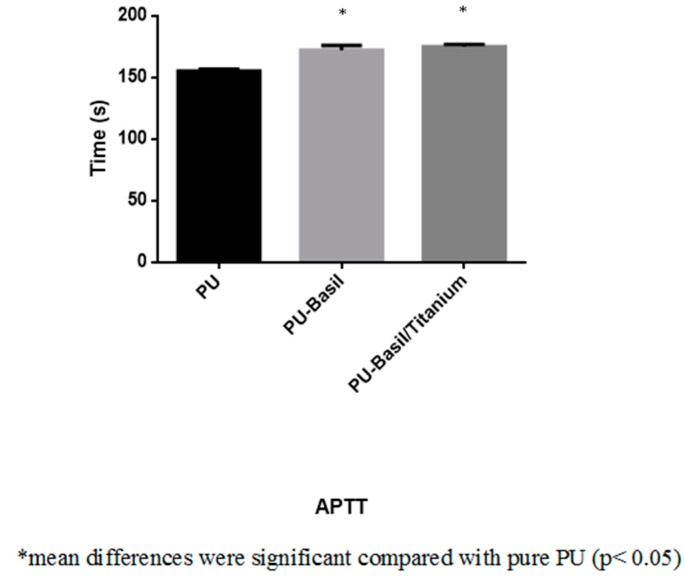
Activated partial thromboplastin time (APTT) assay of PU, PU/BASIL and PU/BASIL/TiO_2_.

**Figure 12:**
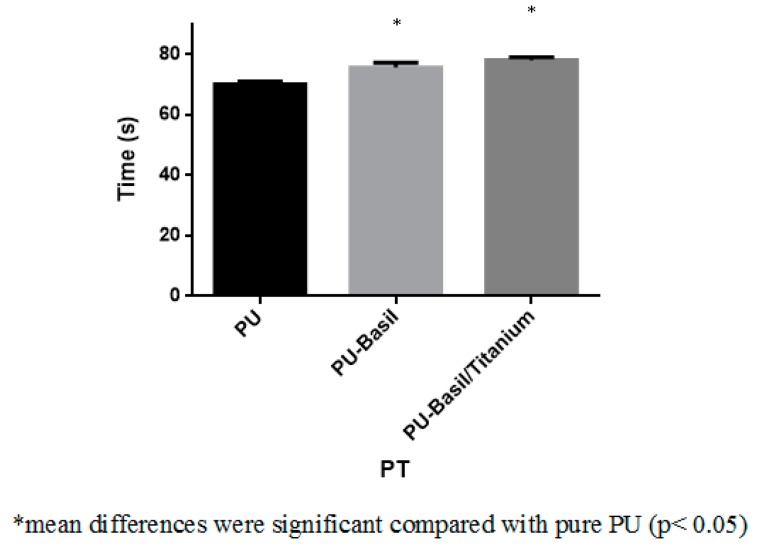
PT assay PU, PU/BASIL and PU/BASIL/TiO_2_.

**Figure 13:**
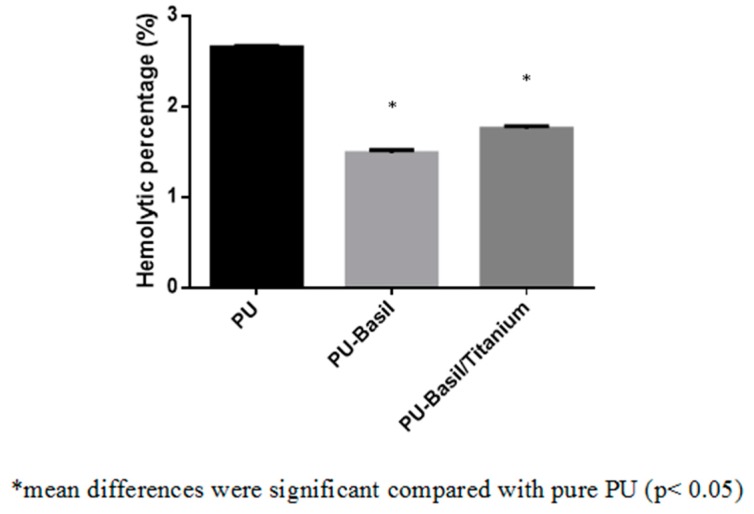
Hemolytic assay PU, PU/BASIL and PU/BASIL/TiO_2_.

The coagulations assays such as APTT and PT was utilized to measure the blood clotting time of the electrospun PU/basil and PU/basil/TiO_2_. The coagulation assay results depicted that the prepared PU/basil and PU/basil/TiO_2_ nanocomposites displayed prolonged clotting time than the pristine PU membrane. The might be due to the addition of basil and TiO_2_ into the PU matrix. The PU/basil and PU/basil/TiO_2_ scaffolds exhibited APTT time of about 172 ± 4 s and 175 ± 2 s, while for PU scaffold, it was only 155 ± 2 s as depicted in Figure 11. Similarly, the PU/basil and PU/basil/TiO_2_ scaffolds exhibited PT time of about 76 ± 2 s and 78 ± 1 s, while for PU scaffold, it was only 70 ± 1 s as depicted in indicated in Figure 12. Next, hemolytic assay was done to determine RBC’s safety with the electrospun membranes. The measurement of hemolytic assay depicted the less hemolytic index for the electrospun PU/basil and PU/basil/TiO_2_ than the Pristine PU. The hemolytic index for the Pristine PU was found to 2.7%, while for the electrospun PU/basil and PU/basil/TiO_2_, the index was reported to 1.49% and 1.75% as indicated in Figure 13. The developed nanocomposites were considered as non-hemolytic because the percentage was below 2% ASTMF756-00(2000) [25,26]. Jaganathan et al. fabricated polyurethane scaffold added with indhulekha oil for tissue engineering applications. The incorporation of indhulekha oil improved the blood clotting time of the Pristine PU. They reported this attribute might due their hydrophobic behavior and smaller fiber diameter [41]. The smaller fiber diameter and hydrophobicity of the developed nanocomposites might have promoted the improved blood compatibility.

### 3.9. Biocompatibility Assessments

**Figure 14:**
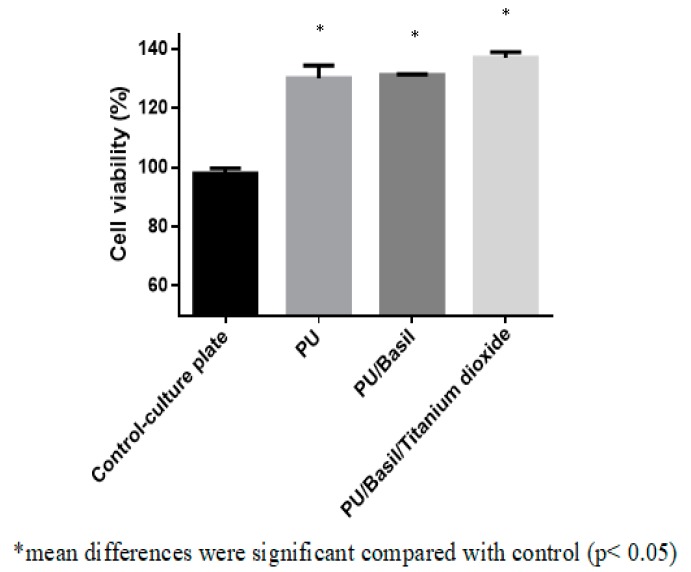
MTS assay PU, PU/BASIL and PU/BASIL/TiO_2_.

The toxicity of the electrospun scaffold with HDF cells was determined using MTS assay and monitored after 5 days culture. After 5 days, the cell viability of the Pristine PU membrane was reported to 130 ± 4 % while the electrospun PU/basil and PU/basil/TiO_2_ displayed cell viability of around 131 ± 0.2 % and 137 ± 2 %as presented in the Figure 14. The electrospun nanocomposites showed marginal increase in the adhesion and proliferation of fibroblast cells compared to the pristine PU. Cell behavior is complex phenomenon and it was influenced by various properties. The properties include fiber diameter, pore size, wettability, roughness, surface chemistry, surface energy, additives, and degradation [25,34,42,43,44,45,46,47,48]. It was reported that the smaller fiber diameter morphology will favors the enhanced cellular response [49]. Our PU/basil and PU/basil/TiO_2_ scaffolds showed smaller fiber diameter than the pristine PU leading to the enhanced cell viability insinuating its potential for cardiac tissue growth. Moreover, the effect of bio-active’s present in the composite and contribution of surface roughness cannot be belittled. 

## 4. Conclusions

This work successfully developed an electrospun novel nanocomposite loaded with basil oil and titanium dioxide (TiO_2_) particles. The composite material displayed increase in hydrophobic and reduced fiber diameter compared to the pristine polymer. Fourier transform infrared spectroscopy results showed the interaction of the pristine polymer with the added substances. Thermal analysis showed the increased onset degradation, whereas the mechanical testing portrayed the increased tensile strength of the composites. Finally, the composite delayed the coagulation times and also rendered safe environment for red blood cells signifying its suitability to be used in contact with blood. Strikingly, the cellular toxicity of the developed composite was lower than the pristine polymer suggesting its compatible nature with the surrounding tissues. With these promising characteristics, developed material with enhanced physicochemical properties and blood compatibility can be successfully utilized for cardiac tissue applications.
